# Case Report: Cerebral lipiodol embolism in the Guangxi Zhuang autonomous region, Southwest China

**DOI:** 10.3389/fmed.2025.1695670

**Published:** 2025-11-04

**Authors:** Wen-Jun Le, Jin-Yu Huang, Zhen-Fang Mao, Zhen-Hua Mo, Hong-Qiao Chen, Wu-Xiao Wei

**Affiliations:** The First Affiliated Hospital of Guangxi University of Science and Technology, Guangxi University of Science and Technology, Liuzhou, China

**Keywords:** cerebral lipiodol embolism, transcatheter arterial chemoembolization, hepatocellular carcinoma, neurological complications, interventional radiology

## Abstract

**Purpose:**

This study aims to provide insights into the rare occurrence of cerebral lipiodol embolism following transcatheter arterial chemoembolization (TACE) in treating hepatocellular carcinoma (HCC). By analyzing a specific case, this research seeks to enhance clinical understanding of the pathogenesis, manifestations, and management strategies for this complication, ultimately improving patient outcomes.

**Background:**

Cerebral lipiodol embolism is an infrequent yet severe complication of TACE, a standard treatment for unresectable HCC. The embolism occurs when iodized oil, used during the procedure, inadvertently enters the cerebral circulation, often due to arteriovenous shunts associated with liver tumors. Despite TACE’s widespread use, awareness and understanding of this rare complication remain limited, necessitating further investigation to mitigate risks and improve patient safety.

**Case presentation:**

A 64-year-old man with multiple HCCs and portal vein invasion underwent TACE involving iodized oil and chemotherapy agents. Post-procedure, the patient exhibited neurological deficits, including decreased consciousness and right-sided weakness. Imaging confirmed cerebral lipiodol embolism. Despite gradual neurological improvement, the patient continued to experience significant right-sided weakness, highlighting the long-term impact of this complication.

**Conclusion:**

Cerebral lipiodol embolism, though rare, poses significant risks during TACE. Early detection through careful imaging and precautionary measures, such as managing Lipiodol injection volumes and speeds, is crucial. Enhanced clinical awareness and intervention strategies can prevent lipiodol from entering the systemic circulation, reducing the incidence of this severe complication.

## Introduction

Primary liver cancer ranks among the most common malignant tumors globally, with persistently high incidence and mortality rates in many countries and regions (1–3). Transarterial chemoembolization (TACE) is internationally established as the standard treatment for unresectable, non-metastatic hepatocellular carcinoma (HCC) and is widely adopted as a first-line local therapy for unresectable liver cancer in China (4–6). The procedure involves the direct delivery of chemotherapy agents into the tumor’s arterial supply, accompanied by embolic materials to occlude tumor vasculature, thereby inhibiting tumor progression and extending patient survival (5, 7, 8). With its broad clinical application, attention to complications has grown, particularly regarding under-recognized rare adverse events. Reported complications of TACE include post-embolization syndrome, sepsis, acute liver failure, liver abscess, intrahepatic bile duct injury, extrahepatic organ embolism, pseudoaneurysm formation, cholecystitis, and tumor rupture (9). Among these, cerebral lipiodol embolism following TACE is exceedingly rare; however, it poses a serious threat to patient survival, highlighting the critical need for meticulous management during interventional therapy for liver cancer. This study highlights the rarity of cerebral lipiodol embolism following TACE, aiming to raise clinical awareness among healthcare professionals. Through these analyses, this study contributes to enhancing physicians’ ability to recognize this rare complication. Such case reports are of significant clinical value in expanding the knowledge base of healthcare providers and improving their alertness to rare diseases.

## Case presentation

**Figure 1:**
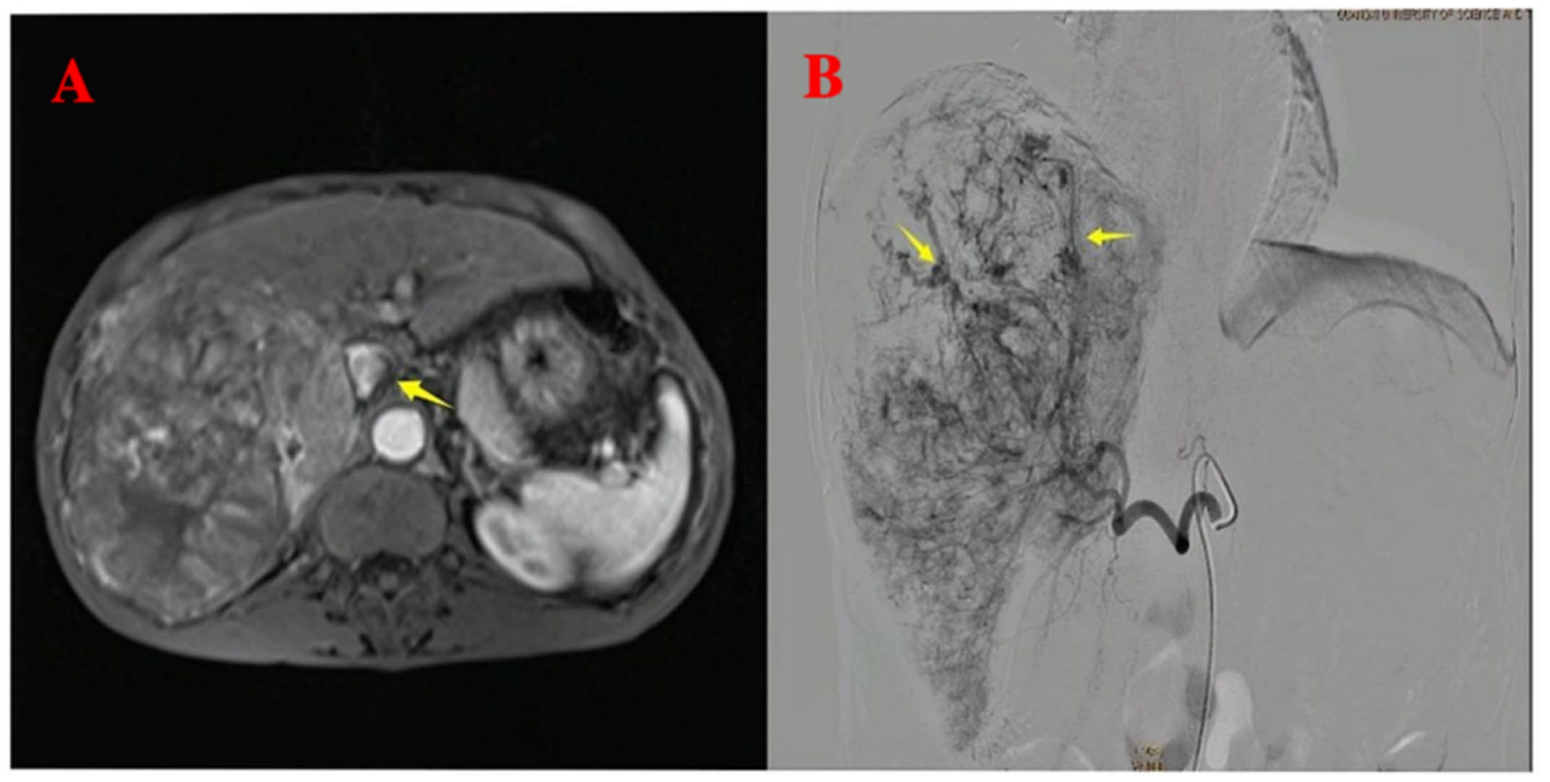
Pre-procedural imaging and intra-procedural angiographic findings. **(A)** Pre-procedural axial T1-weighted contrast-enhanced imaging reveals multiple hepatocellular carcinoma lesions with portal vein invasion (yellow arrow). **(B)** Intra-procedural digital subtraction angiography during the first TACE demonstrates arteriovenous shunting.

**Figure 2:**
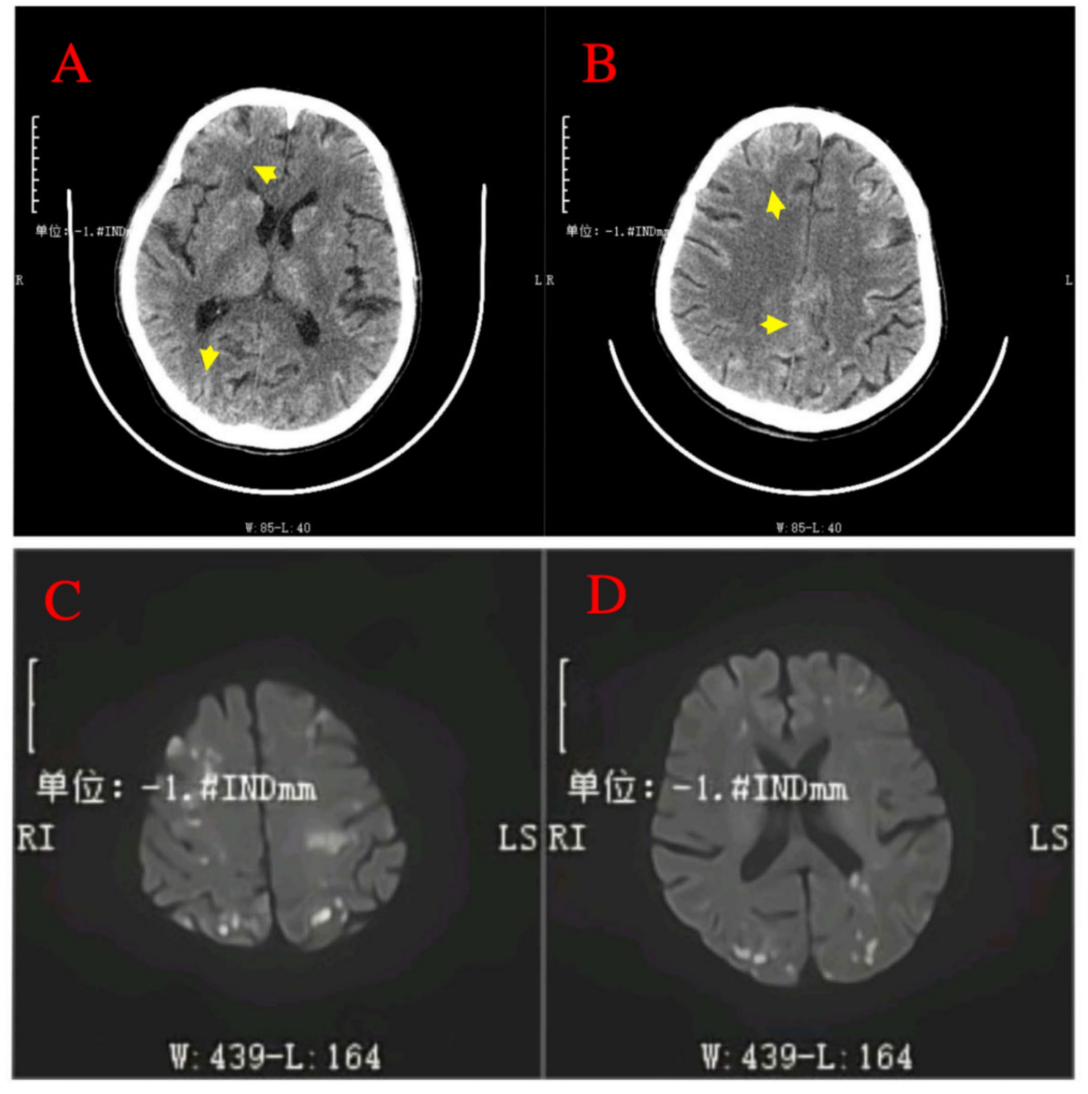
Post-procedural neuroimaging findings of cerebral lipiodol embolism. **(A,B)** Non-contrast head CT scans post-TACE reveal multiple hyperdense foci (indicative of radiopaque Lipiodol deposition) distributed across bilateral cerebral cortices, subcortical regions, basal ganglia, thalamus, and periventricular areas (arrows). **(C,D)** Diffusion-weighted imaging (DWI) MRI sequence performed 24 h after TACE confirms numerous scattered punctate and patchy areas of high signal intensity within the bilateral frontal, temporal, parietal, occipital lobes, and basal ganglia.

A 64-year-old male patient with multiple HCCs and portal vein invasion (Figure 1A) was classified as Child–Pugh B. Preoperative tumor markers showed an alpha-fetoprotein (AFP) level of 12,263.96 ng/mL (reference range: <10.0 ng/mL) and carcinoembryonic antigen (CEA) of 16.4 ng/mL (reference range: <10.0 ng/mL). Laboratory tests revealed a white blood cell count of 9.43 × 10^9^/L (reference range: 4.0–10.0 × 10^9^/L) and platelet count of 195 × 10^9^/L (reference range: 100–300 × 10^9^/L). Liver function tests showed albumin 32.7 g/L (reference range: 40–55 g/L), aspartate aminotransferase (AST) 843.6 U/L (reference range: 15–40 U/L), alanine aminotransferase (ALT) 238.8 U/L (reference range: 9–50 U/L), total bilirubin (TBIL) 34.8 μmol/L (reference range: 0–26 μmol/L), direct bilirubin (DBIL) 14.90 μmol/L (reference range: 0–6.8 μmol/L), and indirect bilirubin (IBIL) 19.9 μmol/L (reference range: 0–17.0 μmol/L). Renal function was normal. Transthoracic echocardiography revealed no atrial septal defect or other intracardiac shunts. The patient underwent the first TACE. Given the tumor’s diameter exceeding 130 mm, the procedure was performed under close monitoring to ensure no reflux of lipiodol. A total of 40 mL of lipiodol, 2 mg of raltitrexed, and 20 mg of lobaplatin were infused. Due to the patient’s family’s limited financial resources and refusal to use coil embolization, gelatin sponge particles (560–710 μm) were used to block the vessels. Following embolization, hepatic arteriography was repeated, confirming the successful closure of the arteriovenous fistula, after which the tumor was embolized with lipiodol. Intraoperative angiography demonstrated an arterioportal shunt (Figure 1B). Twenty hours postoperatively, the patient developed decreased consciousness and right-sided weakness with numbness, without headache or visual disturbances. Physical examination revealed blood pressure of 116/75 mmHg (1 mmHg = 0.133 kPa), with normal pulse, respiratory rate, and body temperature. Neurological examination showed a clear mental status with appropriate responses, right nasolabial fold flattening, midline tongue protrusion, right upper limb muscle strength grade 2, other limbs grade 5, decreased superficial sensation on the right side, and positive Babinski sign on the right; no other pathological signs were noted. Head CT performed 20 h and 30 min postoperatively revealed multiple abnormal density lesions in the bilateral cerebral cortex, subcortical regions, basal ganglia, thalami, and periventricular areas (Figures 2A,B). MRI performed 24 h postoperatively, including diffusion-weighted imaging (DWI), showed scattered punctate and patchy hyperintense or slightly hyperintense lesions in the bilateral frontal, temporal, parietal, and occipital lobes as well as the basal ganglia, with poorly defined margins (Figures 2C,D). Neurology consultation suggested cerebral embolism secondary to iodized oil. The patient’s consciousness gradually improved over the following week; however, right-sided weakness persisted without significant recovery. The patient remains under follow-up.

## Discussion

Cerebral lipiodol embolism is an extremely rare complication following TACE, resulting from the presence of an arteriovenous shunt in liver cancer patients. This abnormal blood vessel pathway allows lipiodol to enter the venous system and form emboli (9). Although TACE has become a standard treatment for hepatocellular carcinoma that is not amenable to surgical resection, the incidence of ectopic cerebral lipiodol embolism in TACE patients is estimated to be <0.01% (10, 11). Cerebral lipiodol embolism was first reported during the early application of traditional tubal and lymphatic angiography techniques, which revealed the significant risk of lipiodol contrast agent migrating to cerebral blood vessels under certain pathophysiological conditions (12–14).

The exact mechanism of lipiodol cerebral embolism remains unclear; however, it is known that hepatic arteriovenous shunts and the unique blood supply characteristics of liver cancer are key factors contributing to this complication (15). Some studies suggest that when the tumor is located near the diaphragm, particularly if it invades the chest cavity or lung base, an abnormal shunt may form between the tumor’s arterial blood supply and the pulmonary arteries or veins (16). This enables lipiodol to enter the systemic circulation through this shunt, potentially causing cerebral lipiodol embolism (16). Additionally, tumor size and location, the amount of lipiodol used, and multiple TACE treatments are considered significant risk factors for the occurrence of cerebral lipiodol embolism (17, 18). In the present case, intraoperative angiography revealed an abnormal arteriohepatic venous shunt along with high-flow, hypervascular tumor characteristics. These findings suggest a plausible pathway for systemic lipiodol dissemination: the lipiodol emulsion entered the hepatic venous circulation via the tumor-associated arteriohepatic venous shunt. Subsequently, it reached the right heart and pulmonary arteries. From there, the lipiodol likely crossed the pulmonary capillary bed either by functional passage of micronized droplets or via a concomitant intrapulmonary or intracardiac right-to-left shunt, ultimately reaching the systemic circulation and the cerebral vasculature (17, 19, 20). Despite prophylactic embolization of the shunt with gelatin sponge particles (560–710 μm)—a measure taken instead of coil embolization due to financial constraints—the relatively large particle size and inherent limitations of this material may have allowed micronized lipiodol droplets to pass through into the venous outflow. Consequently, lipiodol reached the cerebral vasculature, leading to widely distributed emboli. This scenario underscores that, even with embolization of macroscopic shunts, the use of high lipiodol volumes (40 mL in this case) in the presence of high-flow shunts can still result in cerebral embolization, particularly when optimal embolic agents or balloon-occlusion techniques are not utilized. Additionally, while transthoracic echocardiography (TTE) was performed in this case and did not reveal an intracardiac shunt, we acknowledge the limitations of TTE in adults, as it cannot completely exclude the presence of such a shunt. For future cases, we recommend considering transesophageal echocardiography (TEE) or cardiac catheterization as part of the preoperative work-up to more reliably assess for intracardiac shunts, which could further clarify the pathway for systemic embolization.

Patients with cerebral lipiodol embolism exhibit varying onset times and a range of clinical symptoms, primarily including headache, coma, and limb paralysis, all indicative of cerebral ischemia, with symptom severity closely related to the location and extent of the embolism (11, 17, 21). Imaging studies, such as CT and MRI, play a crucial role in diagnosing cerebral lipiodol deposition, typically showing widespread high-density areas or hyperintense regions within the brain parenchyma. This is essential for differentiating from other pathological conditions, such as cerebral hemorrhage (9, 18, 21). Currently, treatment for lipiodol cerebral embolism is primarily supportive and symptom-based, including maintaining airway patency, reducing intracranial pressure, providing neuroprotection, and controlling seizures (10, 17).

The management of cerebral lipiodol embolism is guided by the principle that prevention is paramount over treatment, a strategy that must be systematically implemented throughout the preoperative, intraoperative, and postoperative phases. Comprehensive preoperative evaluation serves as the first critical step. Prior to lipiodol injection, superselective angiography is essential to delineate the tumor-feeding arteries and to actively screen for hepatic arteriovenous shunts. The detection of shunt signs, such as early venous opacification, constitutes the highest risk factor, warranting the strict prohibition of direct lipiodol injection to prevent its entry into the systemic circulation. For patients suspected of having a right-to-left cardiac shunt (e.g., atrial septal defect), further investigation with TEE is recommended; confirmed anomalies necessitate extreme caution or avoidance of lipiodol (19). In patients undergoing repeated TACE sessions, routine assessment of extrahepatic arterial supply is mandatory to prevent lipiodol leakage through collateral pathways (17). Although pre-procedural ^99^ᵐTc-MAA pulmonary perfusion imaging has been proposed for risk prediction, its adoption remains limited due to technical and cost constraints (22). Meticulous intraoperative technique is the cornerstone of prevention. Strict control of the total lipiodol volume (typically ≤20 mL per session), administered via slow, low-pressure injection under continuous fluoroscopic monitoring, is essential to prevent emulsion fragmentation and the formation of dispersible microparticles, while the “sandwich” technique can be used in massive tumors to improve retention (17, 20, 23). If an arteriovenous shunt is confirmed, it must be definitively occluded using agents such as small-caliber gelatin sponge particles or coils before any lipiodol is administered (24). Additionally, balloon-occlusion TACE (B-TACE) can achieve temporary flow arrest in the target vessel, effectively reducing embolic agent dispersion due to hemodynamic washout (25). Vigilant postoperative monitoring is crucial to early intervention. The onset of any neurological symptoms during or after the procedure should raise immediate suspicion for cerebral embolism, prompting urgent cranial imaging for definitive diagnosis and enabling the timely initiation of supportive and symptomatic treatment.

## Conclusion

Although cerebral lipiodol embolism is a rare complication of TACE, it can lead to severe clinical outcomes when it occurs. Careful imaging examination can help identify hepatic arteriovenous shunts and potential blood flow diversions, enabling preventive embolization or interventions before TACE to prevent lipiodol from entering the systemic circulation. Furthermore, appropriately controlling the amount and injection speed of lipiodol, especially during multiple TACE treatments, can effectively reduce the risk of embolism.

## Data Availability

The original contributions presented in the study are included in the article/supplementary material, further inquiries can be directed to the corresponding author.
